# Supplementary material for: From sequence to enzyme mechanism using multi-label machine learning
Source: BMC Bioinformatics. 2014 May 19;15:150. doi: 10.1186/1471-2105-15-150 (PMC4229970; doi:10.1186/1471-2105-15-150)
Supplement: Additional file 2 — Java code of ml2db. Additional file ml2db_code.tar.gz contains the Java source code to run the multi-label machine learning experiments and save the results to database. The code’s Javadoc is included. [file 1471-2105-15-150-S2.zip › additional file 2/ml2db/ml2db/doc/mulan/data/class-use/LabelsMetaData.html]

Uses of Interface mulan.data.LabelsMetaData


JavaScript is disabled on your browser.


- Overview
- Package
- Class
- Use
- Tree
- Deprecated
- Index
- Help

- Prev
- Next

- Frames
- No Frames

- All Classes

## Uses of Interface mulan.data.LabelsMetaData

- Packages that use LabelsMetaData

  | Package | Description |
  | --- | --- |
  |  |  |
  | --- | --- |
  | mulan.classifier.meta |  |
  | mulan.data |  |
- - ### Uses of LabelsMetaData in mulan.classifier.meta

    Methods in mulan.classifier.meta that return LabelsMetaData

    | Modifier and Type | Method and Description |
    | --- | --- |
    |  |  |
    | --- | --- |
    | `LabelsMetaData` | HierarchyBuilder.`buildLabelHierarchy(MultiLabelInstances mlData)` Builds a hierarhy of labels on top of the labels of a flat multi-label dataset, by recursively partitioning the labels into a specified number of partitions. |

    Methods in mulan.classifier.meta with parameters of type LabelsMetaData

    | Modifier and Type | Method and Description |
    | --- | --- |
    |  |  |
    | --- | --- |
    | `static MultiLabelInstances` | HierarchyBuilder.`createHierarchicalDataset(MultiLabelInstances mlData, LabelsMetaData metaData)` Creates the hierarchical dataset according to the original multilabel instances object and the constructed label hierarchy |
  - ### Uses of LabelsMetaData in mulan.data

    Classes in mulan.data that implement LabelsMetaData

    | Modifier and Type | Class and Description |
    | --- | --- |
    |  |  |
    | --- | --- |
    | `class` | `LabelsMetaDataImpl` Implementation of `LabelsMetaData` info about labels and their structure. |

    Methods in mulan.data that return LabelsMetaData

    | Modifier and Type | Method and Description |
    | --- | --- |
    |  |  |
    | --- | --- |
    | `LabelsMetaData` | LabelsMetaDataImpl.`clone()` |
    | `LabelsMetaData` | LabelsMetaData.`clone()` Returns a deep copy of the `LabelsMetaDataImpl` instance. |
    | `static LabelsMetaData` | LabelsBuilder.`createLabels(java.io.InputStream inputStream)` Creates a `LabelsMetaData` instance from specified input stream. |
    | `static LabelsMetaData` | LabelsBuilder.`createLabels(java.lang.String xmlLabelsFilePath)` Creates a `LabelsMetaData` instance from XML file specified by the path. |
    | `LabelsMetaData` | MultiLabelInstances.`getLabelsMetaData()` Gets the `LabelsMetaData` instance, which contains descriptive meta-data about label attributes stored in underlying `Instances` data set. |

    Methods in mulan.data with parameters of type LabelsMetaData

    | Modifier and Type | Method and Description |
    | --- | --- |
    |  |  |
    | --- | --- |
    | `static void` | LabelsBuilder.`dumpLabels(LabelsMetaData labelsMetaData, java.io.OutputStream outputStream)` Dumps specified labels meta-data, in XML format, into the specified `OutputStream`. |
    | `static void` | LabelsBuilder.`dumpLabels(LabelsMetaData labelsMetaData, java.lang.String xmlDumpFilePath)` Dumps specified labels meta-data into the file in XML format. |

    Constructors in mulan.data with parameters of type LabelsMetaData

    Constructor and Description

    `MultiLabelInstances(weka.core.Instances dataSet,
    LabelsMetaData labelsMetaData)`

    Creates a new instance of `MultiLabelInstances` data from existing `Instances`
    and `LabelsMetaData`.

- Overview
- Package
- Class
- Use
- Tree
- Deprecated
- Index
- Help

- Prev
- Next

- Frames
- No Frames

- All Classes
